# N-Acetylcysteine-Amide Protects Against Acute Acrylamide Neurotoxicity in Adult Zebrafish

**DOI:** 10.3390/toxics13050362

**Published:** 2025-04-30

**Authors:** Niki Tagkalidou, Júlia Goyenechea-Cunillera, Irene Romero-Alfano, Maria Olivella Martí, Juliette Bedrossiantz, Eva Prats, Cristian Gomez-Canela, Demetrio Raldúa

**Affiliations:** 1 Institute for Environmental Assessment and Water Research (IDAEA-CSIC), Jordi Girona, 18, 08034 Barcelona, Spain; niki.tagkalidou@idaea.csic.es (N.T.); jbdqam@cid.csic.es (J.B.); 2 Department of Analytical and Applied Chemistry, School of Engineering, Institut Químic de Sarrià-Universitat Ramon Llull, Via Augusta 390, 08017 Barcelona, Spain; juliagoyenecheac@iqs.url.edu (J.G.-C.); ireneromeroa@iqs.url.edu (I.R.-A.); mariaolivellam@iqs-blanquerna.url.edu (M.O.M.); cristian.gomez@iqs.url.edu (C.G.-C.); 3 Research and Development Center (CID-CSIC), Jordi Girona, 18, 08034 Barcelona, Spain; eva.prats@cid.csic.es

**Keywords:** acrylamide, neurotoxicity, zebrafish model, glutathione, acoustic startle response, habituation, kinematic analysis

## Abstract

Acrylamide (ACR) is a potent neurotoxicant that disrupts cellular redox homeostasis by depleting reduced glutathione (GSH) and inducing oxidative stress. Despite its well-characterized mechanism, no effective treatments for ACR-induced neurotoxicity currently exist. This study evaluates the therapeutic efficacy of N-acetylcysteine-amide (AD4), a blood–brain barrier (BBB)-permeable derivative of N-acetylcysteine, in a novel severe acute ACR neurotoxicity model in adult zebrafish. Adult zebrafish received a single intraperitoneal (i.p.) injection of ACR (800 μg/g), followed by AD4 (400 μg/g i.p.) or PBS 24 h later. ACR exposure reduced brain GSH levels by 51% reduction at 48 h, an effect fully reversed by AD4 treatment. Behavioral analyses showed that AD4 rescued ACR-induced deficits in short-term habituation of the acoustic startle response (ASR). Surprisingly, ACR exposure did not alter the neurochemical profile of key neurotransmitters or the expression of genes related to redox homeostasis, synaptic vesicle recycling, regeneration, or myelination. These results demonstrate AD4’s neuroprotective effects against acute ACR-induced brain toxicity, highlighting its therapeutic potential and validating adult zebrafish as a translational model for studying neurotoxic mechanisms and neuroprotective interventions.

## 1. Introduction

Acrylamide (ACR) is a water-soluble type-2 alkene widely used in industrial applications [1,2]. It is known for its mutagenic, carcinogenic, and neurotoxic properties [3]. In humans, ACR exposure typically leads to peripheral neuropathy, characterized by ataxia, skeletal muscle weakness, and sensory deficits in the extremities [4,5,6]. The neurotoxic effects of ACR are primarily attributed to its ability to form adducts with specific proteins critical for maintaining neural homeostasis [7]. For instance, we and others have provided evidence demonstrating that ACR can form adducts with free thiol groups on key components of the antioxidant defense system, such as reduced glutathione (GSH) and thioredoxin, disrupting the cellular redox state by increasing the levels of reactive oxygen species (ROS) [7,8,9].

Zebrafish (*Danio rerio*) has emerged as a powerful vertebrate model for neurotoxicity studies due to its conserved nervous system architecture and neurotransmitter pathways, which closely resemble those of mammals, including humans [10,11]. Both larval and adult zebrafish exhibit neurotoxic responses to ACR exposure, including molecular and behavioral alterations comparable to those observed in mammalian models [9,12,13,14]. While larvae offer high-throughput screening capabilities, adult zebrafish provide a more translationally relevant system for studying complex neurobehavioral outcomes due to their fully developed central nervous system and diverse behavioral repertoire [15].

Despite extensive research into the mechanisms of ACR neurotoxicity, effective treatments are still lacking. Current management is mainly symptomatic, and even mild cases of ACR poisoning often result in incomplete recovery [4]. Given that ACR toxicity involves oxidative stress and GSH depletion, antioxidant-based therapeutic strategies have been explored [16]. N-acetylcysteine (NAC), a precursor of L-cysteine and glutathione, has been investigated as a potential treatment due to its ability to replenish intracellular GSH levels and scavenge electrophilic toxicants. However, despite its effectiveness against ACR neurotoxicity in in vitro models, NAC has shown limited success in protecting against ACR-induced neurotoxicity, largely due to its poor blood–brain barrier (BBB) permeability [14,16,17]. N-acetylcysteine amide (NAC-amide or AD4; hereafter referred to as AD4), a modified derivative of NAC with enhanced lipophilicity, was recently developed as a promising neuroprotective agent able to cross BBB [18,19]. Given its improved pharmacokinetic profile, AD4 represents a compelling candidate for mitigating ACR-induced neurotoxicity [16].

In this study, we developed a severe acute ACR neurotoxicity model in adult zebrafish to evaluate the therapeutic efficacy of AD4. Using a combination of behavioral, biochemical, and molecular approaches, we demonstrate that AD4 fully rescues ACR-induced GSH depletion and mitigates behavioral deficits, highlighting its potential as a therapeutic agent for ACR neurotoxicity.

## 2. Materials and Methods

### 2.1. Chemicals

Acrylamide (CAS 79-06-1) was purchased at Sigma-Aldrich (St. Louis, MO, USA; A9099-25G, purity 100%). N-acetylcysteine-amide (NAC-amide or AD4; CAS 38520-57-9) was a kind gift from Dr. Daphne Atlas, at the Hebrew University of Jerusalem (Israel). Prior to the start of the experiment ACR and AD4 were dissolved in phosphate-buffered saline (PBS) at concentrations 80 mg/mL and 40 mg/mL, respectively.

### 2.2. Fish Husbandry

Wild-type adult zebrafish (blue short-fin phenotype; 0.41 ± 0.1 g) were sourced from Pisciber (Barcelona, Spain). These wild-type zebrafish are genetically heterogeneous and, therefore, more representative of the variability of natural populations than wild-type laboratory strains [20,21]. Zebrafish were acclimated at the Research and Development Center (CID-CSIC) for two months before the start of the experiments. The fish were housed in a recirculating zebrafish system (Aquaneering Inc., San Diego, CA, USA) under controlled conditions. They were kept in 2.8 L tanks filled with fish water, consisting of reverse-osmosis purified water supplemented with 90 mg/L Instant Ocean^®^ (Aquarium Systems, Sarrebourg, France), along with 0.58 mM CaSO_4_·2H_2_O and 0.59 mM NaHCO_3_. Environmental parameters were maintained at 28 ± 1 °C with a 12-h light/12-h dark photoperiod. The zebrafish were fed twice daily with dried food (TetraMin, Tetra GmbH, Melle, Germany).

### 2.3. Experimental Design

All procedures were conducted in compliance with the institutional guidelines and were approved by the Institutional Animal Care and Use Committees at CID-CSIC (OH 1432/2023), with authorization from the local government (agreement number 9820). The study adhered to the ARRIVE guidelines for reporting animal research [22]. For intraperitoneal (i.p.) injections, zebrafish were randomly selected from breeding tanks (0.38–0.45 g; ≈50:50 male:female ratio) and anesthetized via hypothermia. Fish were positioned dorsoventrally, and each individual received an injection of 10 µL of solution per gram of body weight into the peritoneal cavity using a 10 µL glass Hamilton syringe (Hamilton company, Reno, NV, USA) equipped with an ultrafine needle [23]. A total of 156 adult zebrafish were used in this study.

**Figure 1:**
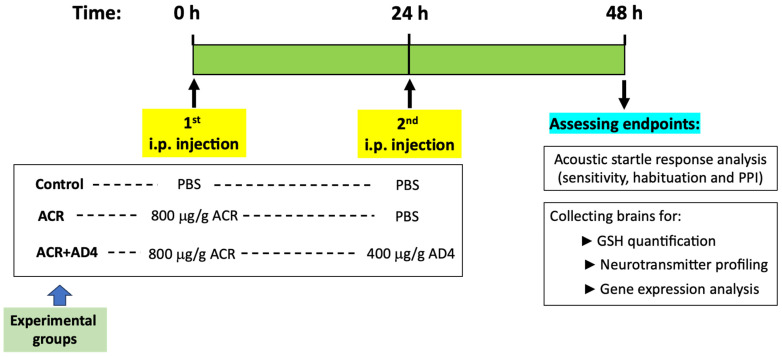
Timeline showing the experimental design of the acute acrylamide (ACR) neurotoxicity model in adult zebrafish. Fish received a first intraperitoneal (i.p.) injection of 800 μg/g b.w. ACR (ACR and ACR+AD4 groups) or PBS (control group) at time 0, followed by a second i.p. injection of PBS (ACR and control groups) or 400 μg/g b.w. AD4 (ACR+AD4 group) at 24 h. At 48 h, kinematic analysis of the acoustic startle response (ASR) and its modulation (sensitivity, habituation and prepulse inhibition (PPI)) was performed, followed by brain collection for GSH quantification, neurotransmitter profiling, and gene expression analysis.

Three experimental groups were set (see Figure 1). The control group received a 1st i.p. injection of PBS at time 0 and a 2nd i.p. injection of PBS at 24 h. ACR group received a 1st i.p. injection of 800 μg/g of ACR at time 0 and a 2nd i.p. injection of PBS at 24 h. Finally, the AD4 group received a 1st i.p. injection of 800 μg/g of ACR at time 0 and a 2nd i.p. injection of 400 μg/g of AD4 at 24 h.

Throughout the study, zebrafish were housed in 2.8 L tanks, provided with food, and maintained in a climate-controlled room at 28 °C under a 12-h light/12-h dark cycle. Water changes were performed daily. At the end of the experimental period, fish were euthanized by hypothermic shock in ice-cold water. Brains were dissected 48 h after the first injection and stored at −80 °C for subsequent analyses.

### 2.4. GSH Determination

A total of 33, 32, and 32 individual brains were analyzed in the control, ACR, and ACR+AD4 experimental groups, respectively, with samples collected from five independent experiments. The quantification of reduced glutathione (GSH) was performed using a fluorescence-based microplate assay, adapted from White et al. (2003) [24]. This method detects GSH by measuring the fluorescence emitted upon its conjugation with naphthalene dicarboxaldehyde (NDA). Briefly, zebrafish brains were weighed before homogenization in ice-cold TES/SB buffer (20 mM Tris, 250 mM sucrose, 1 mM EDTA, 20 mM sodium borate, 2 mM serine, pH 7.4) at a ratio of 16.5 mg/mL. The homogenates were centrifuged at 12,000× *g* for 10 min at 4 °C, and the supernatants were collected for enzyme activity assays and protein quantification. An aliquot of the supernatant (100 µL), referred to as the extract sample, was used for baseline GSH measurement. To inhibit glutamate-cysteine ligase (GCL) activity and precipitate proteins, the extract was diluted threefold by adding equal volumes (100 µL each) of TES/SB buffer and ice-cold 200 mM sulfosalicylic acid (SSA). The mixture was incubated on ice for 10 min to facilitate protein precipitation, followed by centrifugation at 2500× *g* for 5 min at 4 °C.

For fluorescence measurement, 20 µL of the resulting supernatant was transferred in triplicate to a black 96-well microplate (Greiner Bio-One, Germany; flat-bottom, polystyrene, black with clear bottom, catalog no. 655090). The extract was further diluted 2.25-fold (resulting in a total dilution of 6.75-fold) with 50 mM Tris-HCl buffer (pH 10). Next, 155 µL of an NDA alkaline solution (50 mM Tris buffer, pH 10; 0.5 N NaOH; 10 mM NDA in DMSO; *v*/*v*/*v*, 7:1:1) was added to each well, resulting in a total reaction volume of 200 µL per well. The microplate was covered with a matching black lid (Greiner Bio-One) to minimize light exposure and prevent evaporation, and incubated at room temperature for 30 min in the dark. The fluorescence intensity of the GS-NDA cyclic compound was measured using a Synergy 2 Multi-Mode Microplate Reader (BioTek Instruments, Winooski, VT, USA) with excitation and emission wavelengths of 485 nm and 535 nm, respectively. GSH concentrations were determined using a standard curve, and results were expressed as nmol of GSH formed per minute per milligram of tissue.

### 2.5. Neurotransmitters Assessment

Neurotransmitters, their precursors, and degradation products were extracted from adult zebrafish brains following the methodology outlined in Mayol-Cabré et al. [25]. Briefly, six brains from each experimental group were homogenized using a TissueLyser LT (Quiagen, Hilden, Germany), and the resulting supernatant was subjected to centrifugation. The final extract was filtered through a 0.22 μm nylon filter before being transferred directly into chromatographic vials for analysis.

To ensure accurate quantification, an internal standard mixture was added to all samples, allowing for calibration via an internal standard curve. Additionally, quality control (QC) samples were prepared by spiking extracts with a native standard mixture at a concentration of 5 ppm for the target compounds. These QCs were used to assess the efficiency and reliability of the extraction procedure.

The extraction process required high-purity solvents, including HPLC-MS-grade acetonitrile (VWR Chemicals, Leuven, Belgium), LC-MS/MS-grade formic acid (Fisher Scientific, Loughborough, UK), and ammonium formate (Sigma-Aldrich). Ultra-pure water was freshly generated using a Millipore Milli-Q purification system (Millipore, Bedford, MA, USA).

Neurochemical analyses were carried out using UHPLC-MS/MS (Triple Quad 7500 System-QTrap^®^ Ready, SCIEX, Framingham, MA, USA). Analyte separation and elution were performed on a BEH Amide column, while detection was conducted in multiple reaction monitoring (MRM) mode with electrospray ionization in positive mode (ESI+), ensuring high specificity in both detection and quantification.

Quantification was performed using a reference standard mixture containing acetylcholine, tryptophan, 5-hydroxytryptophan (5-HTP), serotonin, 5-hydroxyindole acetic acid (5-HIAA), tyrosine, L-3,4-dihydroxyphenylalanine (L-DOPA), dopamine, 3,4-dihydroxyphenylacetic acid (DOPAC), 3-methoxytyramine (3-MT), norepinephrine (NE), normetanephrine, and epinephrine, all obtained from Sigma-Aldrich. In addition, an internal standard mixture was used for further quantification, comprising DL-norepinephrine-d6 (NE-d6), 3-methoxytyramine-d4 hydrochloride (3-MT-d4), and dopamine-1,1,2,2-d4 hydrochloride (DA-d4). These internal standards were sourced from Sigma-Aldrich and Toronto Research Chemicals (TRC, Toronto, ON, Canada).

### 2.6. Gene Expression Analysis

Total RNA was isolated from whole brains of adult zebrafish from the control, ACR or ACR+AD4 groups sampled 48 h after ACR injection, using Trizol Reagent (Invitrogen Life Technologies, Carlsbad, CA, USA), following previously described protocols [26]. RNA concentration was measured using a NanoDrop™ ND-8000 spectrophotometer (Thermo Fisher Scientific, Waltham, MA, USA). To remove genomic DNA contamination, samples underwent DNase I treatment (Ambion, Austin, TX, USA), after which first-strand cDNA synthesis was performed using 1 μg of total RNA, the First Strand cDNA Synthesis Kit (Roche Diagnostics, Mannheim, Germany), and oligo(dT) primers, according to the manufacturer’s instructions.

Quantitative real-time PCR (qRT-PCR) was carried out using a LightCycler^®^ 480 Real-Time PCR System with SYBR Green PCR Master Mix (Roche Diagnostics) to determine gene expression level. The thermal cycling conditions consisted of an initial denaturation step at 95 °C for 15 min, followed by 45 amplification cycles of 95 °C for 10 s and 60 °C for 30 s.

Each experimental group included nine biological replicates, with three technical replicates per sample. The selection of target genes was based on our previous results on transcriptome analysis after ACR exposure, including genes related to redox homeostasis (*gsr*, *gclc*, *txn*), recycling of synaptic vesicles (*nsf1a*, *syn2a*, *syt1a*, *syt2a*, *stxbp1b*), regeneration-associated genes (*gap43*, *gfap*, *tubb5*) and myelination (*mbp*). The expression of these 12 genes was analyzed alongside the geometric mean of the values for the reference genes 2-peptidylprolyl isomerase A (*ppiaa*) and elongation factor 1a (*elf1a*), with primer sequences provided in Supplementary Table S1. Prior to analysis, primer efficiencies and specificities were verified. Relative mRNA abundance was calculated using the ΔΔCt method [27], with fold-change values derived accordingly.

### 2.7. Kinematic Analysis of the Acoustic Startle Response

Kinematic analyses were conducted on 18 animals from the control, ACR, and ACR+AD4 experimental groups across three independent trials, in a controlled environment (28 °C, darkness) between 10:00 and 17:00. Prior to testing, all zebrafish underwent a one-hour acclimation period in the behavioral testing room.

The kinematic parameters of the acoustic startle response (ASR) in adult zebrafish were evaluated using the Zebra_K platform, as previously described [28]. Additionally, the impact of ACR on sensorimotor gating was assessed through pre-pulse inhibition (PPI) analysis using the same platform. High-speed recordings (115 ms, 1000 fps) were obtained simultaneously from nine experimental arenas using a Photron Fastcam Mini UX100 camera (Photron Ltd., Tokyo, Japan). Video analyses provided key kinematic parameters of the initial C-bend, including latency, duration (time from latency onset to the maximum body curvature), curvature (difference between peak curvature during the C-bend and initial curvature at latency onset), and maximal angular velocity.

The short-term habituation study protocol, which involved analyzing 9 naïve animals from each experimental group, adapted from the methodology described by Wolman et al. (2011) [29] for zebrafish larvae, consists of four distinct phases. Initially, in the sensitivity phase, adult subjects were presented with five audiovisual (AV) stimuli of moderate intensity at 120-s intervals, typically triggering startle responses in 15–30% of cases. Next, during the prehabituation phase, five AV stimuli capable of eliciting startle responses were administered at the same interval. This was followed by the habituation phase, involving 15 consecutive startle-inducing AV stimuli delivered at 1-s intervals. This phase is further divided into early (stimuli 1–5), intermediate (stimuli 6–10) and late (stimuli 11–15) habituation. After a rest period of 300-s, the recovery phase commenced, during which five additional startle-inducing AV stimuli were presented every 120 s. The level of habituation was determined by comparing the average startle responses from the final five stimuli of the habituation phase with those from the prehabituation phase [29].

For PPI assessment, which involved analyzing 9 naïve animals from each experimental group, a low-intensity stimulus (1000 Hz, 10 μs, 72.9 dB re 20 μPa) that typically triggers a 0–10% startle response was used as the prepulse, while a stronger stimulus (1000 Hz, 1 ms, 103.9 dB re 20 μPa) was applied as the startle-inducing pulse. The stimulation protocol consisted of three phases: five prepulse-only stimuli (interstimulus interval [ISI]: 120 s), followed by five pulse-only stimuli (ISI: 120 s), and finally, five combined “prepulse + pulse” sequences (ISI: 120 s). To examine the effect of temporal spacing, two different prepulse-to-pulse intervals (0.5 and 1 s) were tested. PPI percentages were calculated as previously described [30].

### 2.8. Data Analysis

Statistical analysis was performed using GraphPad Prizm v5 (Graph Pad Software, San Diego, CA, USA). The normality of the data was assessed using the Shapiro–Wilk test. Descriptive statistics were presented as mean ± standard error (SEM) for parametric data, and as median and interquartile range (IQR) for non-parametric data. Data from locomotor activity (total distance traveled) and kinematic studies (latency, duration, curvature and maximal angular velocity) were analyzed using an unpaired *t*-test or Mann–Whitney test, with regard to the results of normality distribution. Significance was set at *p* < 0.05. Post hoc power analysis was performed using G*Power 3.1.9.6 software (Heinrich Heine University, Düsseldorf, Germany), with α = 0.05. Values ≥ 0.8 indicate robust sensitivity; 0.4–0.8 suggest moderate detection risk; <0.4 indicates high Type II error potential [31].

## 3. Results

### 3.1. Identification of a Single Dose of Acrylamide Inducing Brain GSH Depletion

The first step in this study was to develop a new severe acute neurotoxicity model induced by ACR in adult zebrafish, based on intraperitoneal administration of this neurotoxicant in a single high dose. Therefore, a range finding test was conducted, injecting fish with 400, 600, 800 and 1000 μg ACR/g body weight (b.w.), and brains were collected 24 h after injection. As shown in Supplementary Figure S1A, the highest decrease in brain GSH levels, about 70%, was found 24 h after injection with 800 μg/g without further enhancing the effect on GSH depletion. The effect of a single i.p. injection on brain GSH levels was further confirmed in a new experiment with a larger sample size (Supplementary Figure S1B). Therefore, a single dose of 800 μg ACR/g b.w. (11.25 mM) was used to build the new model.

### 3.2. AD4 Fully Counteracts the Depletion of GSH Stores in the Brain Led by ACR Exposure

Figure 1 summarizes the experimental design used in this study. Upon evaluating lethality using this experimental design, a mortality rate of 13.7 ± 4.3% (mean ± SE) was found in the ACR-exposed group. In contrast, no lethality was observed in either the control or the ACR+AD4 co-treatment groups during the study.

**Figure 2:**
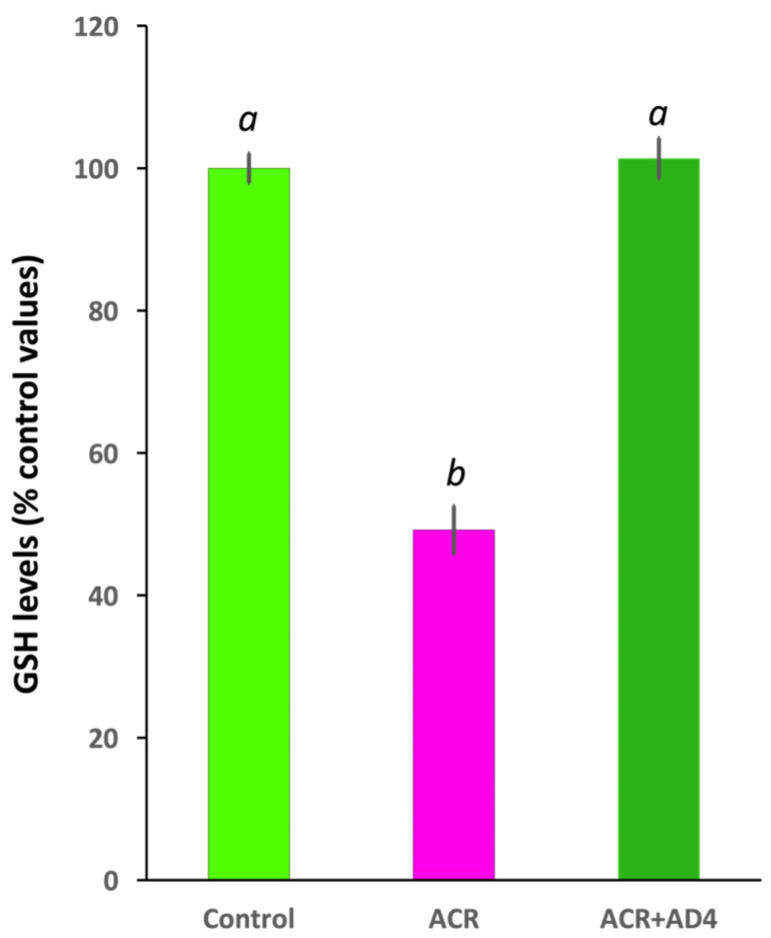
N-acetylcysteine-amide (AD4, single dose, 400 μg/g b.w., i.p.) administered 24 h after acrylamide exposure (single dose, ACR, 800 μg/g b.w., i.p) leads to full recovery of the reduced glutathione (GSH) levels in the adult zebrafish brain. Different letters indicate significant differences (*p* < 0.05) following one-way ANOVA (N: 23–33).

Using this model we found that 48 h after the ACR injection, GSH levels decreased to 51.46 ± 3.01% of control values [*N*_control_ = 33, *N*_ACR_ = 32 (5 independent experiments); *t*(63) = 14.014, *p* = 4.99 × 10^−21^]. AD4 [400 μg/g b.w. (2.50 mM)], administered intraperitoneally 24 h after ACR injection, was able to fully counteract the ACR-induced depletion of brain GSH stores (Figure 2, Supplementary Dataset S1). While GSH in the brains of ACR-injected fish decreased to 49.19 ± 3.38% (*N* = 26) of controls, administration of AD4 24 h after ACR injection led to a full recovery, with brain GSH levels 101.38 ± 2.85% (n = 23) of the control (*F*(2,73) = 113.548, *p* = 3.90 × 10^−23^). GSH levels in the brain of the ACR-treated fish were significantly lower than those of control and ACR+AD4 treated fish (ACR vs. Control, *p* = 5.10 × 10^−9^; ACR vs. ACR+AD4, *p* = 5.10 × 10^−9^; Control vs. ACR+AD4, *p* = 0.937; one-way ANOVA with Tukey’s multiple comparison test; *N*  =  23–27, from 4 independent experiments). The post-hoc power analysis for GSH comparisons between groups showed a power (1-β) of ≥0.8.

### 3.3. Neurochemical Profile in the Brain of ACR-Injected Zebrafish Remains Unaltered

ACR exposure has been reported to induce changes in the neurotransmitter profile in the brain of different animal models. In contrast, when the profile of neurotransmitters was analyzed in the brain of control and ACR-injected fish, no changes were found in the levels of acetylcholine (*t*(10) = 0.277, *p* = 0.788), serotonin (*t*(10) = −1.292, *p* = 0.225), dopamine (*t*(10) = −0.942, *p* = 0.369) or norepinephrine (*t*(10) = −0.441, *p* = 0.668) (Supplementary Table S2). No differences were also found in the precursors of serotonin (tryptophan: *t*(10) = 0.305, *p* = 0.767; 5-HTP: *t*(10) = −0.767, *p* = 0.461) and dopamine (tyrosine: *t*(10) = −0.627, *p* = 0.545; L-DOPA: *t*(10) = −0.031, *p* = 0.976) as well as in the degradation products of serotonin (5-HIAA: *t*(10) = −1.794, *p* = 0.103), dopamine (DOPAC: *t*(10) = 0.394, *p* = 0.704; 3-MT: *t*(10) = 0.103, *p* = 0.920; HVA: *t*(10) = −0.356, *p* = 0.729) and norepinephrine (normetanephrine: *t*(10) = 1.299, *p* = 0.223; epinephrine: *t*(10) = 0.681, *p* = 0.511).

Post-hoc power analysis indicated low statistical power (<0.4) for all neurochemical comparisons.

### 3.4. Lack of Differential Gene Expression in the Brain of ACR-Injected Zebrafish

Waterborne exposure to ACR has been reported to induce changes in the expression of genes related to redox homeostasis, synaptic vesicle recycling, regeneration, and myelination in the brain of adult zebrafish [8,9]. However, as shown in Supplementary Table S3, no significant differences were observed in the expression of 12 selected genes from these functional groups in the brain of zebrafish 48 h after i.p. injection of ACR. Specifically, the expression levels of *nsf1a*, *syn2a*, *syt1a*, *syt2a*, *stxbp1b*, *gsr*, *gclc*, *txn*, *gap43*, *tubb5*, *gfap*, and *mbp* did not differ between control and ACR-injected fish. The post-hoc power analysis of the gene expression data showed that *nsf1a* and *syn2a* exhibited a statistical power above 0.8, while *syt2a*, *stxbp1b*, *gclc*, *gap43*, *tubb5*, *gfap*, and *mbp* had power between 0.4 and 0.8. In contrast, *gsr* and *txn* had power below 0.4.

### 3.5. ACR Delays Habituation to a Series of Acoustic Startle Stimuli and This Effect Is Counteracted by AD4

In previous studies, we demonstrated that waterborne exposure to ACR leads to anxiety-like behavior in adult zebrafish [9,13]. However, we found that intraperitoneal injections lead to relevant changes in this type of behavior, suggesting that anxiety is not a suitable apical endpoint to be used in this study. Instead, in this work, we evaluated the effect of ACR on the sensitivity, habituation and prepulse inhibition of the acoustic startle response (ASR).

As shown in Table 1 and Supplementary Dataset S2, ACR injection did not impair the main kinematic parameters of the ASR, including the latency, duration of the bending, amplitude of the C-bend, average angular velocity and maximal angular velocity of the ASR. Moreover, Supplementary Dataset S3 shows that no differences in sensitivity to the startle stimulus have been found between control (20.00 ± 4.16% of responses) and ACR-injected (22.22 ± 3.51% of responses) fish (*t*(8) = −0.408, *p* = 0.694). For the prehabituation step of the protocol, the frequency of responses for ACR-treated fish (*Mdn* = 55.56%) showed a trend to decrease with respect to the corresponding controls (*Mdn* = 66.67%), and AD4 administration was not able to rescue this effect (*Mdn* = 55.56%). While the Kruskal–Wallis test revealed a statistically significant difference among the three groups (*H*(2, 15) = 7.335, *p* = 0.026), pairwise comparisons failed to identify significant differences among groups after correction for multiple comparisons (Control vs. ACR, *p* = 0.057; Control vs. ACR+AD4, *p* = 0.057; ACR vs. ACR+AD4, *p* = 1.00).

**Figure 3:**
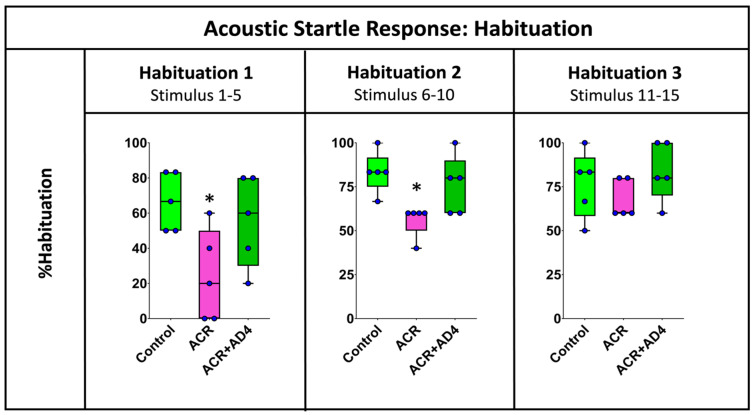
Analysis of the % habituation in adult zebrafish control, ACR and ACR+AD4 during the initial (stimuli 1–5), middle (stimuli 6–10) and final (stimuli 11–15) part of the habituation step (series of 15 startle stimuli with a 1 s interstimulus interval). Boxplot representation with the box indicating the 25th and 75th percentiles and the whiskers the maximum and minimum values. The thin line within the box marks the median. * *p* < 0.05 (N: 9 fish per experimental group, 15 reactions, in total, per fish).

Figure 3 shows that ACR injection delays habituation to the ASR and that this effect is fully recovered when AD4 is administered. The effect of ACR on the habituation process was analyzed by determining the % habituation during the initial (stimuli 1–5), middle (stimuli 6–10) and final (stimuli 11–15) periods of this neuromodulatory process (Supplementary Dataset S3). Figure 3 shows statistical differences between groups during the first five stimuli (*F*(2,12) = 4.516, *p* = 0.036), with a clear decrease in the % habituation in animals injected with ACR (*Mdn* = 20%) with respect to the corresponding controls (*Mdn* = 66.67%), and a full recovery of this parameter after the administration of AD4 (*Mdn* = 66.67%). A similar profile was found during the second period of habituation (stimuli 6–10; *H*(2,15) = 8.519, *p* = 0.014), with % habituation of 83.33%, 60.00% and 83.00% for the control, ACR-injected and ACR+AD4 injected groups, respectively. Finally, during the last period of the habituation (stimuli 11–15), a similar trend was also observed, but this time the differences were not statistically significant (*H*(2,15) = 2.559, *p* = 0.278).

Finally, when % PPI (500 and 1000 ms as prepulse-to-pulse interval) of ASR was determined in adult zebrafish from the control, ACR, and ACR+AD4 groups, no statistically significant differences were found between control and ACR-treated fish (Supplementary Dataset S4).

## 4. Discussion

In previous studies, we demonstrated that acute waterborne exposure to 0.75 mM ACR for 72 h resulted in a 56% depletion of brain GSH levels (about 56%) in adult zebrafish, which was not rescued by NAC due to its limited blood–brain barrier (BBB) permeability [9,13,14]. In this study, we developed a novel severe acute ACR neurotoxicity model using intraperitoneal (i.p.) injections to evaluate the therapeutic potential of AD4, a BBB-permeable derivative of NAC [32]. However, administering the antidote via water exposure at concentrations ranging from 300 to 750 µM would require gram-scale amounts of the compound. While this approach is feasible for NAC due to its relatively low cost, AD4 is over 7000 times more expensive, making this exposure route impractical. Therefore, the first step in this study was to develop a novel severe acute acrylamide-induced neurotoxicity model in adult zebrafish based on intraperitoneal injections. The results presented in this manuscript demonstrate that 48 h after a single dose of 800 μg ACR/g b.w., brain GSH levels decreased by approximately 51%, an effect comparable to the 56% reduction observed in the previous waterborne exposure model [9].

The limited BBB permeability of NAC [33] is evident when comparing brain GSH recovery between NAC [14] and AD4 (this study) in ACR-exposed animals. In the former study, animals were pretreated for 24 h with two concentrations of NAC (equimolar and 0.4 times the ACR concentration) before being co-exposed to a mixture of 0.75 mM ACR and NAC at the same concentrations used for pretreatment, for an additional 72 h. In contrast, in the present study, AD4 was not administered before or during ACR exposure but only once 24 h after ACR administration. Additionally, the dose of AD4 was only 0.22 times the ACR dose. Remarkably, a full recovery of brain GSH levels was observed in this study with AD4, whereas no effect was found in the previous work with any concentration of NAC [14].

The disruption of synaptic vesicle recycling is a well-documented mechanism of ACR neurotoxicity, mediated by covalent modification of thiolate sites on proteins critical for synaptic function [34,35,36]. In our earlier work, waterborne ACR exposure altered the expression of genes involved in synaptic vesicle cycling (e.g., *nsf1a*, *syt1a*, *syn2a*, *stxbp1b*), redox homeostasis (e.g., *gsr*, *gclc*, *txn*), and regeneration (e.g., *gap43*, *gfap*, *tubb5*) in zebrafish brains [8,9,12]. However, in the current i.p. model, no significant changes were observed in the expression of these genes. This discrepancy may reflect differences in exposure paradigms: continuous waterborne exposure likely induces sustained cellular stress and transcriptional responses, whereas a single high-dose i.p. injection may cause rapid GSH depletion without triggering prolonged oxidative or neuroinflammatory signaling. Moreover, the post-hoc power analysis for the gene expression data showed that only two genes exhibited a statistical power above 0.8, indicating a high likelihood of detecting true effects. Ten genes had power between 0.4 and 0.8, suggesting moderate sensitivity, while two genes had power below 0.4, implying a high risk of Type II errors. These findings highlight variability in the robustness of the statistical analysis across genes and suggest that some results should be interpreted with caution.

Similarly to the gene expression results, while waterborne ACR exposure altered neurotransmitter profiles in zebrafish brains [9], the i.p. model did not affect the levels of acetylcholine, serotonin, dopamine, or norepinephrine. These findings suggest that the toxicokinetics of ACR exposure play a critical role in shaping its neurotoxic effects, as acute i.p. exposure primarily targets redox homeostasis without inducing broader transcriptional or neurochemical changes. Similarly to the observed effect at gene expression level, the discrepancy may stem from differences in toxicokinetics: continuous waterborne exposure likely induces sustained cellular stress, while a single high-dose intraperitoneal injection may cause rapid GSH depletion without triggering prolonged transcriptional or neurochemical responses [37]. Additionally, the resilience of neurotransmitter systems in our model could reflect compensatory mechanisms that maintain neurochemical balance under acute chemical stress conditions [38]. While these results suggest that neurotransmitter dynamics remain stable under the current exposure paradigm, further studies with finer temporal resolution or region-specific analyses are needed to fully rule out transient or localized effects. Finally, it is important to note that a key limitation of the neurotransmitter analysis in this study is the low statistical power (0.05 to 0.368), indicating a high probability of Type II errors. This suggests that the sample size may have been insufficient to detect true differences between groups. Future studies should incorporate a priori power analyses to determine appropriate sample sizes and enhance sensitivity.

Whereas acrylamide is primarily considered a neurotoxic compound affecting the peripheral nervous system, it also impacts the central nervous system, though research in this area remains limited [7]. In this study, we analyzed the effect of acute exposure to ACR on two neuroplasticity processes, short-term habituation and PPI of the ASR. The observed impairment in habituation may be linked to the reported effect of ACR on synaptic vesicle recycling, a process critical for synaptic plasticity and neurotransmitter availability [39].

Granato et al., (2019) [40] identified five habituation regulatory modules in zebrafish, with Module 1 comprising of Huntingtin-interacting protein 14 (Hip14), NMDA receptors and dopamine receptors. Hip14 is a palmitoyl transferase that attaches palmitate to specific cysteine residues of synaptic proteins including receptors, channels, and proteins involved in synaptic vesicle dynamics [40]. By forming adducts with these cysteine residues, ACR could competitively inhibit palmitoylation, disrupting the trafficking and function of synaptic proteins. This mechanism provides a plausible explanation for the observed deficits in habituation, as palmitoylation is essential for synaptic plasticity. Furthermore, ACR’s impairment of synaptic vesicle recycling could reduce the release of glutamate and dopamine into the synaptic cleft, diminishing the activation of NMDA and dopamine receptors. These disruptions would collectively contribute to the observed decrease in habituation.

The impairment in ASR short-term habituation observed in our study also aligns with previous reports linking GSH depletion to deficits in synaptic plasticity [41]. Animal models of schizophrenia and pharmacological GSH depletion (e.g., buthionine sulfoximine-treated rodents) have been shown to exhibit impaired synaptic plasticity and altered habituation responses [41,42]. Given that habituation is a form of non-associative learning requiring efficient synaptic adaptation, the observed deficits in our study may be attributed to oxidative stress-induced disruptions in neural circuits that mediate ASR modulation. Moreover, the fact that ACR did not significantly alter ASR kinematic parameters or % PPI suggests that GSH depletion selectively disrupts neuroplasticity mechanisms underlying habituation without affecting the basic startle reflex or sensory gating processes, as recently reported in rodents [42].

Finally, while AD4 has demonstrated compelling neuroprotective efficacy in this study, its current cost (around 150 EUR/5 mg) presents a significant translational challenge. This cost primarily reflects small-scale experimental synthesis [19] and a lack of optimized industrial production. Historical precedents suggest that scaling up synthesis (e.g., via continuous-flow chemistry or enzymatic methods) could reduce costs by 10–100× [43,44]. Moreover, for acute neurotoxicity treatments where BBB penetration is critical (e.g., acrylamide poisoning or stroke), the cost–benefit ratio may justify targeted use despite premium pricing. Future research should prioritize cost-efficient synthesis routes and clinical scenarios where AD4’s unique BBB permeability offsets economic constraints.

## Figures and Tables

**Table 1:** Kinematic parameters of the C-bend during the acoustic startle response in control and ACR-treated adult zebrafish.

Parameter	Control [Median, (IQR)]	Acrylamide [Median, (IQR)]	*U*	z	*p*
Latency (ms)	11 (10–12)	11 (10–12)	1729	0.347	0.729
Duration (ms)	12 (11–14)	11 (10–13)	1392	−1.556	0.120
Curvature (°)	99.3 (86.6–112.0)	102.5 (77.5–115.8)	1696	0.147	0.883
Average Angular Velocity (°/ms)	8.4 (7.6–9.3)	8.8 (7.7–10.0)	1898	1.267	0.205
Maximal Angular Velocity (°/ms)	17.6 (15.5–19.7)	18.4 (16.2–20.9)	1906	1.311	0.190

U: Mann–Whitney U statistic; z: z-score.

## Data Availability

The data supporting the findings of this study are available within the manuscript and its Supplementary Materials file or will be made available from the corresponding author upon request.

## Supplementary Materials

The following supporting information can be downloaded at: https://www.mdpi.com/article/10.3390/toxics13050362/s1, Supplementary Figure S1: Effect of the intraperitoneal injection with a single dose of different concentrations of acrylamide (ACR) on GSH levels in the brain of adult zebrafish 24 h; Supplementary Table S1: List of primers used for the qPCR; Supplementary Table S2: Levels of acetylcholine and monoaminergic neurochemicals (pg/mg tissue) in the brain of vehicle-injected control, ACR-injected and ACR+AD4-injected adult zebrafish; Supplementary Table S3: Gene expression analysis of 12 selected genes in the brain of adult zebrafish, either control or injected with 800 μg ACR/g ww; Supplementary Dataset S1: Brain reduced glutathione levels in adult zebrafish i.p. injected with PBS/PBS (control group), acrylamide/PBS (ACR group) and acrylamide/AD4 (ACR+AD4 group); Supplementary Dataset S2: Kinematic parameters of the C-bend during the acoustic startle response in control and ACR-treated adult zebrafish; Supplementary Dataset S3: Effect of acrylamide on short-term habituation of the acoustic startle response and the potential recovery by using AD4; Supplementary Dataset S4: Effect of acrylamide on sensorimotor gating (prepulse inhibition) of the acoustic startle response and the potential recovery by using AD4.

## Abbreviations

The following abbreviations are used in this manuscript:

AD4	N-acetylcysteine-amide
ACR	Acrylamide
ASR	Acoustic Startle Response
GSH	Reduced Glutathione
NAC	N-acetylcysteine
