# Supplementary material for: N-Acetylcysteine-Amide Protects Against Acute Acrylamide Neurotoxicity in Adult Zebrafish
Source: Toxics. 2025 Apr 30;13(5):362. doi: 10.3390/toxics13050362 (PMC12115520; doi:10.3390/toxics13050362)
Supplement: Supplementary file 1 [file toxics-13-00362-s001.zip › Supplementary Material.pdf]

Supplementary Material

N-Acetylcysteine-Amide Protects Against Acute Acrylamide Neurotoxicity in Adult Zebrafish

Niki Tagkalidou, Júlia Goyenechea-Cunillera, Irene Romero-Alfano, Maria Olivella Martí, Juliette Bedrossiantz, Eva Prats, Cristian Gomez-Canela and Demetrio Raldúa *

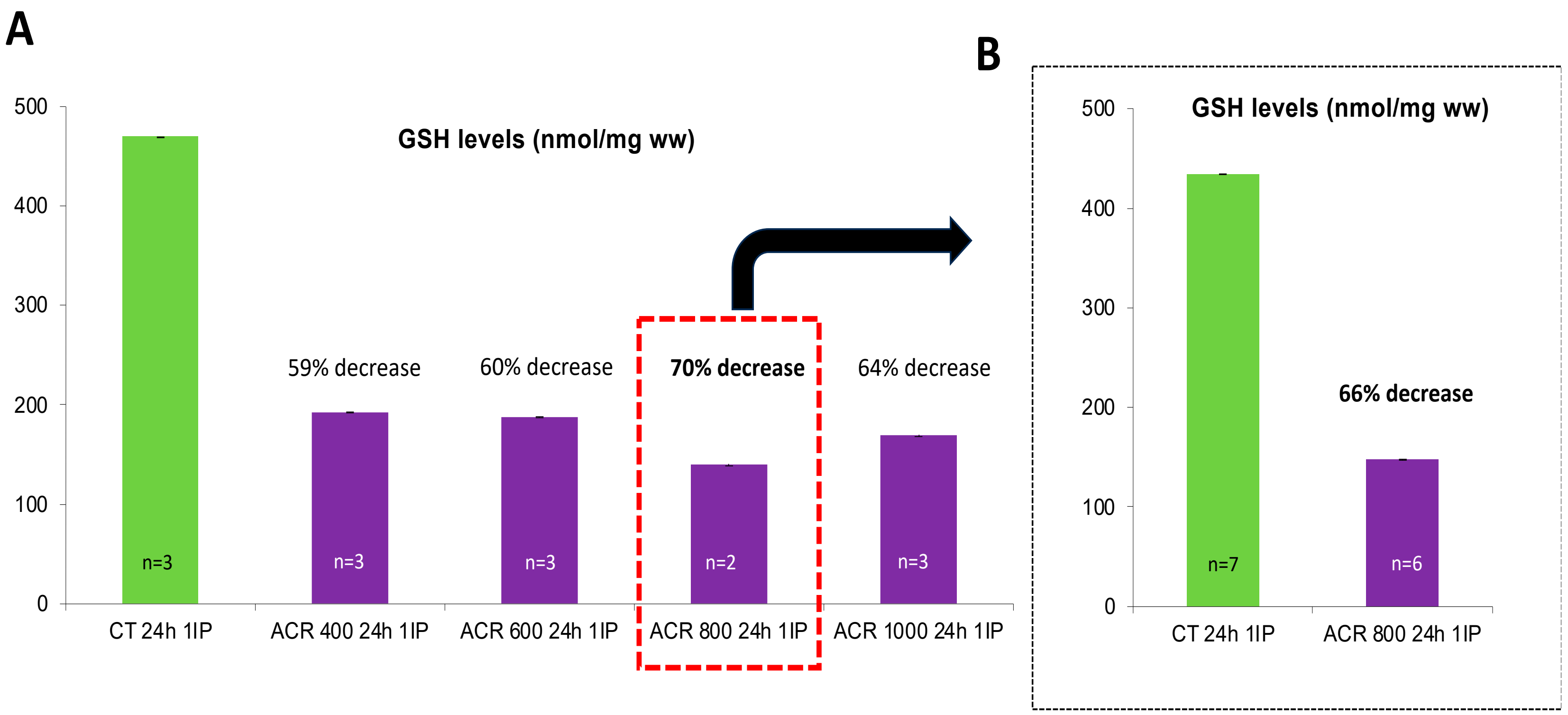

Supplementary Figure S1. Effect of the intraperitoneal injection with a single dose of different concentrations of acrylamide (ACR) on GSH levels in the brain of adult zebrafish 24 h. (A) Range finding test performed for identifying the ACR concentrations resulting in the highest decrease in GSH levels; (B) In the range finding test 800 μg ACR/g b.w. was selected, but the sample size was very low, so a new experiment was performed with this dose but increasing the sampling size (n=6-7). Data presented as mean ± SE

Supplementary Table S1. List of primers used for the qPCR

Gene	ZFIN Acc number	GenBank Acc number	Sequence		Amplicon length
gap43a	ZDB-GENE-990415-87	NM_131341.1	FW	5'-CAGCCGACGTGCCTGAA	71 bp
			RV	5'-GGATTCCTCAGCAGCGTCTG	
gclc	ZDB-GENE-030131-5056	NM_199277.2	FW	5'-AAGTGGATGAGGGAGTTTGTTGCC-3'	90 bp
			RV	5'-CTTGTGGAGCAGGTCGTAGTTGAT-3'	
gfap	ZDB-GENE-990914-3	NM_131373	FW	5'-GGATGCAGCCAATCGTAAT	97 bp
			RV	5'-TTCCAGGTCACAGGTCAG	
gsr	ZDB-GENE-050522-116	NM_001020554.1	FW	5'-CGGCCTCAACCTCAGTCAAA-3'	142 bp
			RV	5'-TGCTTCATCAGGTGTCAGAAGG-3'	
mbp	ZDB-GENE-030128-2	AY860977	FW	5'-AATCAGCAGGTTCTTCGGAGGAGA	102 bp
			RV	5'-AAGAAATGCACGACAGGGTTGACG	
nsf1a	ZDB-GENE-030616-37	NM_001044328.1	FW	5'-CGCGGCTTCTTCGAGTAACA	134 bp
			RV	5'-GAAGTGTGATCTCCGTCAGGTT	
ppiaa	ZDB-GENE-030131-8556	NM_212758.1	FW	5'-GGGTGGTAATGGAGCTGAGA	179 bp
			RV	5'-AATGGACTTGCCACCAGTTC	
syn2a	ZDB-GENE-040718-341	NM_001002597	FW	5'-GTGACCATGCCAGCATTTC	80 bp
			RV	5'-TGGTTCTCCACTTTCACCTT	
syt1a	ZDB-GENE-040718-165	NM_001327829	FW	5'-AAAGGGAAGAGACGGCTGTG	130 bp
			RV	5'-GGAGCCAGGCAGAAGCTTTA	
stxbp1b	ZDB-GENE-060531-166	NM_001089376.1	FW	5'-ACGCTGAAAGAGTACCCAGC	118 bp
			RV	5'-CTCCCAAAGTGGGGTCATCC	
tuba1b	ZDB-GENE-030822-1	NM_194388.2	FW	5'-AATCACCAATGCTTGCTTCGAGCC	117 bp
			RV	5'-TTCACGTCTTTGGGTACCACG	
tubb5	ZDB-GENE-031110-4	NM_198818.1	FW	5’-TGACCTCCAGGGGTCTGA-3’	81 bp
			RV	5’-GTTGGCGTCCAAGTGAGG-3'	
txn	ZDB-GENE-040718-162	NM_001002461.1	FW	5'-AGTTGGTGGTGGTGGACTTC-3'	122 bp
			RV	5'-CACATCCACCTTTAGAAACACC-3'	

Supplementary Table S2. Levels of acetylcholine and monoaminergic neurochemicals (pg/mg tissue) in the brain of vehicle-injected control, ACR-injected and ACR+AD4-injected adult zebrafish

	Control	ACR	ACR+AD4	One-way ANOVA	*P* value
**Acetylcholine**	502.8	591.9	648.5	*F*(2,15) = 0.67	0.936
	1077.5	982.2	709.7		
	779.1	796.8	830.9		
	977.3	842.0	706.2		
	854.8	905.5	1079.3		
	849.2	760.9	871.3		
Mean± SE	840.1 ± 80.2	813.2 ± 54.7	807.7 ± 64.2		

	Control	ACR	ACR+AD4	One-way ANOVA	*P* value
**Tryptophan**	9813.8	7973.9	7307.4	*F*(2,15) = 2.15	0.154
	8937.0	6340.0	9765.7		
	7856.0	7689.8	7775.9		
	5690.1	7017.3	7897.1		
	6850.0	5976.2	11505.3		
	7152.3	9790.1	10883.4		
Mean± SE	7716.5 ± 607.8	7464.6 ± 559.7	9189.1 ± 725.1		

	Control	ACR	ACR+AD4	One-way ANOVA	*P* value
**5-HTP**	203.3	209.7	330.6	*F*(2,15) = 0.53	0.601
	340.6	322.4	300.6		
	371.8	364.2	248.0		
	229.9	291.4	254.9		
	275.8	268.9	538.1		
	306.1	486.5	370.8		
Mean± SE	287.9 ± 26.4	323.9 ± 38.8	340.5 ± 43.8		

Supplementary Table S2 (continued)

	Control	ACR	ACR+AD4	One-way ANOVA	*P* value
**Serotonin**	70.1	106.9	129.1	$F(2,15) = 1.87$	0.189
	295.1	222.8	152.0		
	122.5	230.4	229.0		
	164.4	281.3	150.0		
	232.9	235.5	199.9		
	176.5	328.7	121.9		
Mean± SE	176.9 ± 32.5	243.3 ± 30.3	163.6 ± 17.2		

	Control	ACR	ACR+AD4	One-way ANOVA	*P* value
**5-HIAA**	511.2	580.2	555.1	$F(2,15) = 1.02$	0.383
	569.5	599.0	584.1		
	571.6	573.2	583.6		
	456.4	483.6	494.0		
	499.6	602.5	716.6		
	563.1	654.3	461.4		
Mean± SE	528.6 ± 19.2	582.1 ± 22.9	565.8 ± 36.3		

	Control	ACR	ACR+AD4	One-way ANOVA	*P* value
**Tyrosine**	24142.3	24101.2	30418.7	$F(2,15) = 0.56$	0.584
	34542.3	26235.8	24383.6		
	22008.7	20241.8	26738.7		
	21947.6	35390.4	22330.2		
	23823.4	29535.6	46062.2		
	26991.8	28746.3	26905.2		
Mean± SE	25576.0 ± 1944.4	27375.2 ± 2111.0	29473.1 ± 3498.0		

Supplementary Table S2 (continued)

	Control	ACR	ACR+AD4	One-way ANOVA	*P* value
**L-DOPA**	1064.4	1795.3	1131.7	$F(2,15) = 0.19$	0.827
	1840.6	1943.2	1229.3		
	2566.5	1741.0	1286.0		
	2433.7	2088.4	2752.1		
	2656.5	2208.2	1885.7		
	1005.9	1849.8	2229.4		
Mean± SE	1927.9 ± 305.4	1937.6 ± 73.6	1752.4 ± 265.9		

	Control	ACR	ACR+AD4	One-way ANOVA	*P* value
**Dopamine**	84.7	117.3	163.3	$F(2,15) = 0.60$	0.564
	344.8	238.4	243.0		
	193.9	206.1	316.7		
	169.2	314.3	210.2		
	238.7	267.3	270.4		
	244.6	474.6	209.6		
Mean± SE	217.7 ± 35.5	269.7± 49.1	235.5 ± 21.9		

	Control	ACR	ACR+AD4	One-way ANOVA	*P* value
**DOPAC**	48.7	107.3	100.5	$F(2,13) = 0.34$	0.717
	135.7	180.1	205.5		
	114.9	45.4	295.2		
	73.9	122.7	67.0		
			41.3		
	270.1	101.1	201.6		
Mean± SE	128.7 ± 38.5	111.3 ± 21.6	151.8 ± 40.0		

Supplementary Table S2 (continued)

	Control	ACR	ACR+AD4	One-way ANOVA	*P* value
**3-MT**	190.7	206.6	189.7	$F(2,15) = 0.03$	0.968
	162.2	197.7	291.1		
	155.0	142.1	178.3		
	233.8	222.9	117.0		
	205.5	191.4	196.8		
	258.2	231.8	194.4		
Mean± SE	200.9 ± 16.4	198.8 ± 12.9	194.5 ± 22.8		

	Control	ACR	ACR+AD4	One-way ANOVA	*P* value
**Norepinephrine**	453.9	678.4	521.0	$F(2,15) = 0.16$	0.854
	830.7	647.9	606.6		
	574.3	590.4	659.3		
	609.3	1066.7	718.1		
	848.4	735.2	846.3		
	908.5	775.3	859.7		
Mean± SE	704.2 ± 74.6	749.0 ± 68.8	701.8 ± 54.7		

	Control	ACR	ACR+AD4	One-way ANOVA	*P* value
**Normetanephrine**	284.6	264.1	218.1	$F(2,15) = 1.53$	0.249
	361.4	310.6	244.7		
	133.1	145.6	135.3		
	210.9	251.5	219.6		
	372.4	196.3	301.9		
	331.0	164.0	165.7		
Mean± SE	282.2 ± 38.3	222.0 ± 26.1	214.2 ± 24.0		

Supplementary Table S2 (continued)

	Control	ACR	ACR+AD4	One-way ANOVA	*P* value
**Epinephrine**	16.2	14.9	15.4	$F(2,15) = 0.37$	0.696
	17.9	16.0	14.4		
	22.6	14.1	22.6		
	9.2	11.8	9.7		
	13.2	13.3	14.4		
	19.4	19.3	27.1		
Mean± SE	16.4 ± 1.9	14.9 ± 1.0	17.3 ± 2.6		

Supplementary Table S3. Gene expression analysis of 12 selected genes in the brain of adult zebrafish, either control or injected with 800 µg ACR/g ww. Brains were collected 48 hours after ACR or saline injection. Gene expression levels were calculated using the Log_2 ΔΔCT method, with normalization to the housekeeping genes, *ppia* and *elf1a*. Data are presented as mean ± standard deviation. Statistical analysis was performed using Student's t-test, with no significant differences observed between groups ($p > 0.05$).

	nsf1a	***syn2a***	***syt1a***	***syt2a***	***stxbp1b***	***gsr***	***gclc***	***txn***	***tubb5***	***gap43***	***gfap***	***mbp***
**Control**	-1.406	-0.138	-0.162	2.858	0.005	0.073	0.684	-0.330	0.408	0.429	-0.071	1.097
	0.728	-0.587	-0.270	2.030	-0.453	0.455	0.372	0.478	0.196	0.531	-0.798	0.985
	0.546	0.015	-0.299	-0.680	0.575	-0.642	-1.691	-0.365	-1.131	-1.479	0.319	-1.665
	0.187	0.159	-0.170	-0.458	-1.070	-0.347	-1.453	-0.547	-0.766	-1.861	-0.116	-1.267
	-0.520	-0.204	0.055	2.577	0.302	0.795	0.659	0.318	0.119	0.307	0.080	0.615
	-0.609	0.142	0.181	2.208	-0.085	-0.734	0.450	-0.177	0.351	1.162	0.586	-0.103
	0.792	0.574	0.521	2.920	0.341	0.401	0.979	0.625	0.823	0.911	-0.452	0.338
	0.282	0.040	0.143		0.386						0.451	
**Acrylamide**	-0.883	-0.220	0.263	2.260	-1.027	1.108	0.615	0.508	0.249	0.591	-0.786	0.722
	0.219	-0.101	0.331	0.250	-0.072	-0.705	-1.248	0.151	-0.424	-1.596	0.159	-1.362
	-0.116	-0.243	0.210	0.955	-0.057	-0.205	-0.931	-0.102	-0.681	-1.273	-0.349	-1.765
	-0.056	-0.610	-0.115	-0.003	-0.454	-0.180	-0.710	-0.144	-0.192	-0.548	-0.441	-0.987
	-0.406	-1.076	-0.070	-0.223	-0.810	-0.659	-0.915	-0.659	-0.321	-1.056	-0.646	-1.415
	-1.000	0.054	0.132		0.382	-0.332	-1.295	0.685	-1.311	-2.029	0.029	-1.945
	-1.144	0.124	0.341		0.378						0.853	
	-1.641	-1.211	-0.589		-0.287						-0.777	
	-0.196	-0.416	0.086		0.130						-0.142	
**Student's *t* test**												
t	1.723	2.045	-0.469	1.248	0.806	0.469	1.434	-0.277	1.291	1.653	0.960	1.961
df	15	15	15	10	15	11	11	11	11	11	15	11
P value	0.105	0.059	0.646	0.240	0.433	0.648	0.179	0.787	0.223	0.127	0.352	0.076
